# Graphene−Perfluoroalkoxy Nanocomposite with High Through-Plane Thermal Conductivity Fabricated by Hot-Pressing

**DOI:** 10.3390/nano9091320

**Published:** 2019-09-15

**Authors:** Xinru Zhang, Xiaoyu Xie, Xinzhi Cai, Zeyi Jiang, Ting Gao, Yujie Ren, Jian Hu, Xinxin Zhang

**Affiliations:** 1 School of Energy and Environmental Engineering, University of Science and Technology Beijing, Beijing 100083, China; 2 Beijing Engineering Research Center of Energy Saving and Environmental Protection, University of Science and Technology Beijing, Beijing 100083, China; 3 Beijing Key Laboratory for Energy Saving and Emission Reduction of Metallurgical Industry, University of Science and Technology Beijing, Beijing 100083, China; 4 China Energy Conservation and Environmental Protection Group, Beijing 100082, China; 5 China Energy Conservation and Environmental Protection Group National Machinery United Electric Power (Ningxia) Co., Ltd., Yinchuan 750011, China

**Keywords:** thermal conductivity, nanocomposite, graphene, alignment, hot-pressing, perfluoroalkoxy

## Abstract

With the rapid development of electronics and portable devices, polymer nanocomposites with high through-plane thermal conductivity (TC) are urgently needed. In this work, we fabricated graphene nanosheets−perfluoroalkoxy (GNs−PFA) composite sheets with high through-plane TCs via hot-pressing followed by mechanical machining. When the GNs content exceeded 10 wt%, GNs were vertically aligned in the PFA matrix, and the through-plane TCs of nanocomposites were 10–15 times higher than their in-plane TCs. In particular, the composite with 30 wt% GNs exhibited a through-plane TC of 25.57 W/(m·K), which was 9700% higher than that of pure PFA. The composite with 30 wt% GNs was attached to the surface of a high-power light-emitting diode (LED) to assess its heat-dissipation capability. The composite with vertically aligned GNs lowered the LED surface temperature by approximately 16 °C compared with pure PFA. Our facile, low-cost method allows for the large-scale production of GNs–PFA nanocomposites with high through-plane TCs, which can be used in various thermal-management applications.

## 1. Introduction

Thermal management has become one of the most critical challenges in the design of electronic components, smart phones, light-emitting diodes (LEDs), touch panels, etc. [1,2]. The development of high-power electronic devices needs to obtain new materials with high thermal conductivity, excellent thermal stability, tailored thermal expansion and reduced weight. To address this issue, many novel materials have been developed, such as, polymer matrix composite, carbon matrix composite and metal matrix composite [1,3,4]. Among them, polymers have several favorable attributes for thermal management because of their low cost, light weight, high stability and good processability. Unfortunately, almost all polymers possess low thermal conductivity, on the order of 0.1–0.5 W/(m·K) [5], providing insufficient heat dissipation for effective thermal management. Therefore, improving the thermal conductivity (TC) of polymers is necessary for their application in thermal management.

Over the past two decades, to improve the TC of polymers, extensive studies have been conducted on incorporating nanofillers with high TCs into polymer matrix, such as copper/silver, silicon carbide, and aluminum oxide nanoparticles, carbon nanotubes, graphene nanosheets (GNs), boron nitride nanosheets (BNNs) [6,7,8,9,10,11]. Among these nanofillers, 2-dimensional (2D) platelet-like GNs and BNNs are particularly promising owing to their ultrahigh and unique anisotropic TCs [12,13,14]. For example, previous studies reported that GNs and BNNs had in-plane TCs of 2000−5000 and 600 W/(m·K), respectively, whereas their through-plane TCs ranged from 10 to 20 W/(m·K) [15,16].

Taking advantage of this ultrahigh TC of GNs and BNNs along the in-plane direction, significant efforts have been devoted to enhancing the nanocomposite’s TC by aligning GNs and BNNs in the polymer matrix [13]. Several methods to fabricate composites with aligned GNs or BNNs have been proposed, such as liquid crystal method [17,18], exerting an electric/magnetic field [19,20], vacuum filtration [21,22], unidirectional freeze casting [23,24], and electrospinning [25]. For example, Kumar et al. used a liquid-crystal method to fabricate composite films with layer-aligned GNs in polyvinylidene fluoride co-hexafluoropropylene, and reported that the composite film had an in-plane TC of 19.5 W/(m·K) at a GNs content of 27.2 wt% [26]. By exerting an external magnetic field, Yan et al. aligned GNs in epoxy by functionalizing them with Fe_3_O_4_ nanoparticles, and found that for a GNs content of 0.52 vol%, the TC of the composite was 0.361 W/(m·K) along the direction of GNs alignment [27]. Using the vacuum filtration method, Li et al. fabricated layer aligned GNs–epoxy composite with an in-plane TC of 16.75 W/(m·K) at a GNs content of 11.8 wt% [22]. Using the freeze casting method, Lian et al. fabricated vertically aligned and interconnected GNs–epoxy nanocomposite with through-plane TC of 2.13 W/(m·K) for a GNs content of 0.92 vol% [28].

Recently, based on a facile hot-pressing method, Sun et al. fabricated the polycarbonate composite films with layer aligned BNNs via pre-coating followed by hot-pressing, achieving a TC of 3.09 W/(m·K) for 18.5 vol% BNNs [29]. In addition, Yu et al. fabricated a composite film with layer aligned BNNs in thermoplastic polyurethane via solution ball-milling followed by hot-pressing. They reported TCs of 50.3 and 6.9 W/(m·K) along the in-plane and through-plane directions, respectively, with 95 wt% layer-aligned BNNs [30]. The above studies indicate that it is promising to fabricate the high-performance polymer nanocomposites with well aligned nanosheets by using this facile and low-cost hot-pressing method. Whereas, it should be noted that these examples are 2D films that exhibit high in-plane TCs, because the alignment direction of BNNs is parallel to the surface of composite film. By contrast, in many thermal management applications, composites with high through-plane TCs are more desired [23,27,28]. However, to date, little research has been reported on nanocomposites with high through-plane TCs using the hot-pressing method.

In this work, we fabricated the 3D bulk perfluoroalkoxy (PFA) composites with highly layer-aligned GNs using the hot-pressing method. We then cut the bulk GNs–PFA composites into thin sheets along the direction perpendicular to the alignment direction of GNs. By characterizing the microstructure and TC of these obtained nanocomposite sheets, we found that, when the GNs content exceeded 10 wt%, the GNs had a high degree of vertical alignment and the through-plane TCs were approximately 10–15 times higher than the in-plane TCs. In particular, for a GNs content of 30 wt%, the through-plane TC was 25.57 W/(m·K), which was 9700% higher than that of pure PFA. Our method is facile and low cost, and enables the large-scale production of GNs–PFA nanocomposites with high through-plane TC.

## 2. Materials and Methods

### 2.1. Preparation and Characterization of Graphene Nanosheets (GNs)

The GNs were produced by exfoliating flaked graphite (TNGNP, Chengdu Organic Chemicals Co. Ltd., Chengdu, China) in ethanol–water mixture (ethanol: 45 vol%) using a tip sonicator (Scientz-950E, Scientz Biotechnology Co. Ltd., China) at 300 W for 120 min (see Supplementary Materials (SM) Figure S1 for further details). Then the GNs were collected and redispersed in ethanol (Sinopharm Chemical Reagent Co. Ltd, Shanghai, China) [31,32]. The morphology, thickness and crystal structure of the GNs were then characterized. Specifically, the morphology was observed using a scanning electron microscopy (SEM) (Nova NanoSEM 430, FEI, Hillsboro, OR, USA) operating at a 5-kV acceleration voltage by pipetting the GNs dispersions onto a Si substrate. The GNs thickness was examined using transmission electron microscopy (TEM, JEM-2200FS, JEOL Ltd., Tokyo, Japan) by pipetting the GNs dispersions onto carbon-coated copper grids, atomic force microscope (AFM, Dimension FastScan, Brucker Ltd., Santa Barbara, CA, USA) by pipetting the GNs dispersions onto mica, as well as Raman spectroscopy (LabRAM HR800, Horiba Jobin-Yvon, Lyon, France) with a 514 nm wavelength laser. The crystal structure of GNs was determined by the high-resolution TEM and fast-Fourier transformation.

### 2.2. Fabrication of Aligned Graphene Nanosheets−Perfluoroalkoxy (GNs–PFA) Nanocomposites

**Figure 1:**
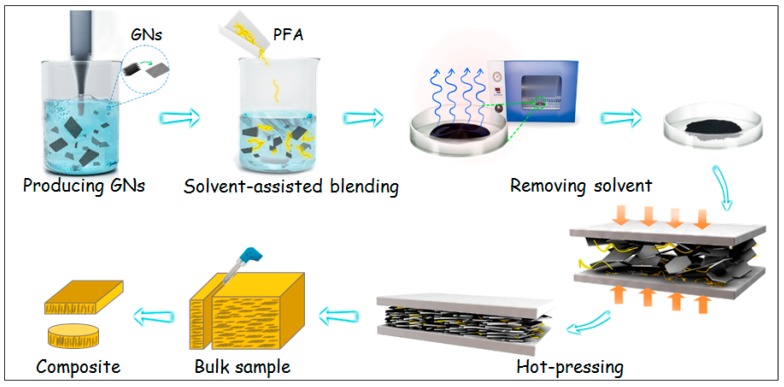
Schematic representation of the fabrication process for the perfluoroalkoxy (PFA) composite with vertically aligned graphene nanosheets (GNs). First, GNs were prepared by exfoliating the flaked graphite. Then, PFA powder was added into the GNs dispersion and mixed while removing the ethanol. Afterwards, the GNs–PFA slurry was dried in a vacuum oven to remove the residual ethanol. The obtained powder was placed in a mold and hot-pressed into the bulk sample. Finally, the bulk sample was cut, perpendicular to its top surface, into thin sheets.

Figure 1 shows the method to fabricate the aligned GNs–PFA nanocomposites via hot-pressing followed by mechanical machining. First, GNs were prepared by exfoliating graphite in an ethanol (45 vol%)–water mixture, followed by redispersion in ethanol. PFA powder (obtained from DuPont, USA) was then added into the GNs dispersions and mixed while removing ethanol under heating and magnetic stirring for 6 h at 60 °C. Afterwards, the GNs–PFA slurry was dried at 120 °C for 24 h in a vacuum oven to remove the residual ethanol. The obtained GNs–PFA powder was put into a mold and heated at 400 °C for 1 h. The molten GNs–PFA sample was hot-pressed at 380 °C under a pressure of 15 MPa for 15 min, then cooled to room temperature under the ambient pressure to form bulk composites (see Figure S2 for the details of the hot-pressing system) [33]. Finally, the bulk composites (length × width × height: 30 × 30 × 11 mm^3^) were cut into many thin sheets (30 × 11 × 1 mm^3^) along the direction perpendicular to the top surface of the bulk composite. The properties of these sheets were then evaluated. We performed the above steps for GNs–PFA composites with GNs contents of 1, 5, 10, 15, 20, 25 and 30 wt%.

### 2.3. Characterization of GNs–PFA Nanocomposite

The thermal stability, microstructure, through-plane and in-plane TCs, heat dissipation capability, as well as coefficient of thermal expansion (CTE) of the GNs–PFA composite sheets were characterized. The thermal stability of the composites was determined by thermogravimetric analysis using a thermal analysis platform (Labsys Evo, Setaram, France) at a heating rate of 10 °C/min from 25 °C to 900 °C under a nitrogen atmosphere. The microstructures of the GNs–PFA composite sheets were observed using SEM (Nova NanoSEM 430, FEI, Hillsboro, OR, USA) operating at a 20-kV acceleration voltage. We note that, prior to the SEM observation, the composites were fractured in liquid nitrogen to form a neat cross-section.

The through-plane TCs (i.e., λ_⊥_) and in-plane TCs (i.e., λ_∥_) of the composite sheets were determined by λ = *α* × *c_p_* × *ρ*, where, *α* is the thermal diffusivity, *c_p_* is the specific heat, and *ρ* is the density of composite sheet. The values of *α* and *c_p_* of the composites were measured using a laser flash thermal analyzer (LFA 427, Netzsch, Germany), and *ρ* was determined by measuring the weight and dimensions of the samples.

To evaluate the application potential of the GNs–PFA composite sheets, we measured their heat dissipation capability when attached to a high-power LED. Specifically, the back surface of the LED was attached to the composite sheet (diameter: 10 mm; height: 1 mm), and a heat sink was connected to the bottom side of the composite. After the LED was switched on, the temperature of the LED was measured using an infrared thermal imaging instrument (E60, FLIR Systems, USA). We compared the heat dissipation capability of the PFA composite with 30 wt% GNs with that of pure PFA.

In addition, the CTE of the composites was measured using an optical dilatometry (DIL 806, TA Instruments, USA), which is capable of measuring, without sample contact, dimensional changes in the sub-micro range. The measurement was conducted in the temperature range from 25 to 200 °C under a 10 °C·min^−1^ heating rate.

## 3. Results and Discussion

### 3.1. Properties of the GNs Produced by Liquid Phase Exfoliation

**Figure 2:**
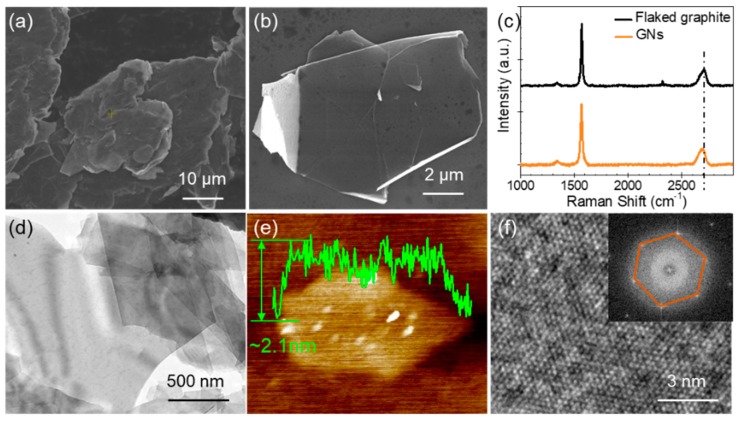
(**a**) SEM image of pristine flaked graphite. (**b**–**f**) SEM image, Raman spectra, bright-field TEM image, AFM image, high-resolution TEM, respectively, of the GNs produced by exfoliating graphite. Inset in (**f**) is fast-Fourier transformation pattern of the GNs.

The morphology, thickness, and crystal structure of the produced GNs were characterized using SEM, Raman spectroscopy, TEM and AFM. As shown in the SEM image in Figure 2a, the flaked graphite had a size of 30–50 μm, and a thickness of several micrometers. Figure 2b shows the SEM image of the GNs produced by exfoliating graphite in ethanol (45 vol%)–water mixture. The GNs had a size of about 5–10 μm. The Raman spectra of the pristine graphite and the GNs produced by exfoliating graphite are shown in Figure 2c. The 2D band (~2750 cm^−1^) of the GNs shifted to a lower position than that of graphite, which demonstrates that the GNs were few-layered [34]. In addition, the bright-field TEM image of the GNs (Figure 2d) indicates that the GNs were few-layered. The AFM image (Figure 2e) shows that the thickness of GNs was approximately 2.1 nm. Evidently, both the TEM and AFM observation were consistent with the Raman spectra measurement shown in Figure 2c. The high-resolution TEM images of the GNs are shown in Figure 2f. The lattice spacing of approximately 0.21 nm suggests that there was no structural distortion for these GNs [35]. The inset of Figure 2f shows the fast-Fourier transformation pattern of the GNs, which demonstrates that the GNs maintained a six-fold symmetry pattern, which is the typical crystal structure of GNs. Together, the characterization of the morphology, thickness, and crystal structure of GNs demonstrated that they had a large size, were few-layered, and had high structural quality.

### 3.2. Thermal Stability of the GNs–PFA Nanocomposites

**Figure 3:**
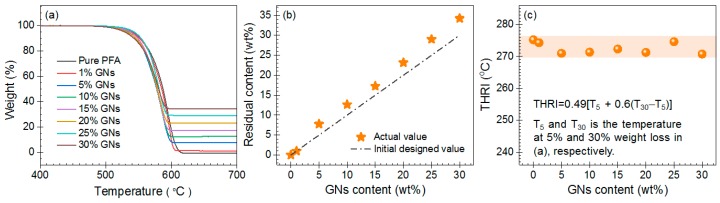
(**a**) Thermogravimetric analysis curves for pure PFA and GNs–PFA nanocomposites. (**b**) The relationship between the initial and residual GNs content obtained in the thermogravimetric analysis. (**c**) The heat resistance index (THRI) for pure PFA and the GNs–PFA nanocomposites.

The thermal stability is an important parameter of nanocomposites. We measured the thermogravimetric analysis curves to analyze the thermal stability of the composites. As shown in Figure 3a, the pure PFA and GNs–PFA nanocomposites showed a similar one-step decomposition between 500 and 620 °C. This result indicates that the addition of GNs had little influence on the decomposition behavior of the PFA composite. Figure 3b shows the relationship between the initial GNs content and the actual residual content obtained in thermogravimetric analysis. Because the GNs are stable at 800 °C in nitrogen, the residual GNs can be used to estimate the GNs content in PFA matrix. The residual content agreed well with the initial GNs content, implying that the GNs were dispersed uniformly in the PFA matrix.

The heat-resistance index (THRI) was used to analyze the thermal stability of the nanocomposite (see Table S1 for details) [36]. As shown in Figure 3c, the THRI value for the pure PFA was about 275.2 °C, whereas those of the nanocomposites was slightly lower (271.0 to 274.6 °C). This result indicates that the addition of GNs slightly reduced the thermal stability of the nanocomposite. This could be due to the ultrahigh TC of GNs, which would serve to increase heat conduction, thus accelerating the decomposition of the matrix [37]. The addition of GNs to the PFA may also decrease the thermal stability by increasing the number of defects in the nanocomposite [38].

### 3.3. Microstructure of the GNs–PFA Nanocomposites

**Figure 4:**
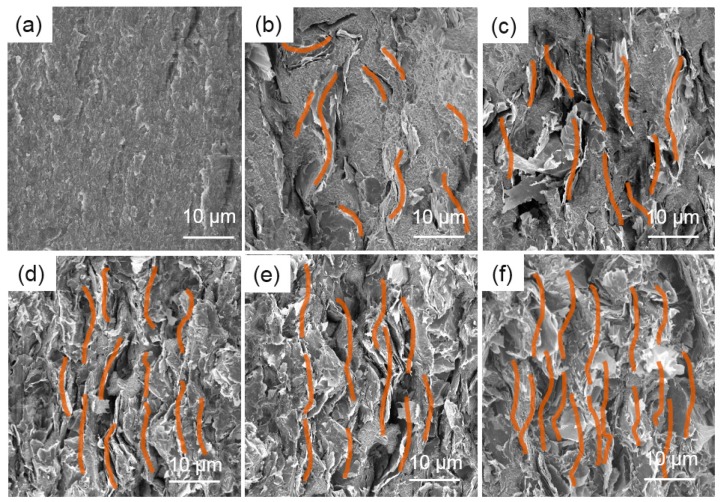
SEM images of the fractured surfaces for pure PFA (**a**), and (**b**–**f**) GNs–PFA nanocomposites containing 5, 10, 15, 20, and 30 wt% GNs, respectively.

The microstructure of the GNs–PFA nanocomposites and the alignment of GNs in PFA were observed using SEM. Figure 4 shows the SEM images of the fractured surfaces for pure PFA, and GNs–PFA nanocomposites containing 5, 10, 15, 20 and 30 wt% GNs. The image in Figure 4a shows the smooth surface of the pure PFA matrix. In the nanocomposite with 5 wt% GNs (Figure 4b), the GNs were well dispersed in the PFA matrix and were almost isolated from each other; however, the GNs were almost randomly ordered, with only a small number vertically aligned. At 10 wt% GNs (Figure 4c), there was clear alignment of GNs in the PFA matrix; however, few GNs were in contact with each other, which means there were few thermally conductive paths in the composite. At 15% GNs (Figure 4d), there were a large number of GNs, nearly all of which were vertically aligned with a high degree of overlap. This contact and overlap of well-ordered, stacked GNs would lead to the formation of thermally conductive paths in the nanocomposite, which is one of the most important factors for improving the TC [39]. At higher GNs contents of 20 and 30 wt% (Figure 4e,f), there was even greater alignment and a higher number of thermally conductive paths than at 10 wt% GNs, which may be due to the increased compactness of GNs in the polymer matrix [40]. In summary, when the GNs content was low (i.e., 5 wt%), there was no clear alignment of GNs in the PFA. At higher content (i.e., 10 wt%), GNs were well aligned, and above 15 wt%, the vertically aligned GNs were in contact with each other, forming thermally conductive paths in the PFA matrix.

### 3.4. Through-Plane and in-Planes Thermal Conductivity (TC) of the GNs–PFA Nanocomposites

**Figure 5:**
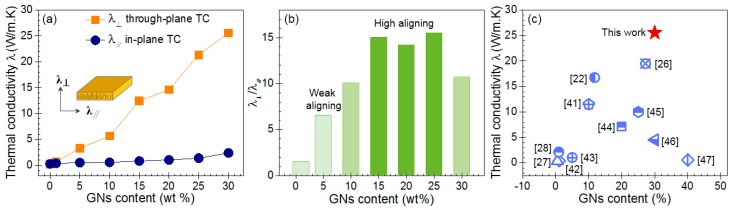
(**a**) Through-plane TCs (λ_⊥_) and in-plane TCs (λ_∥_) of the PFA composites with vertically aligned GNs. (**b**) Ratio of through-plane TC to in-plane TC (*i.e.*, λ_⊥_/λ_∥_). (**c**) Comparison of the through-plane TC of the composite sheet with 30 wt% GNs with the TC values of the GNs-based composites reported in literature.

Figure 5a shows the through-plane (λ_⊥_) and in-plane (λ_∥_) TCs of pure PFA and the GNs–PFA composite sheets with GNs contents of 1, 5, 10, 15, 20, 25, and 30 wt%. The results indicate that pure PFA had a low TC of 0.27 W/(m·K). At GNs contents higher than 10 wt%, the through-plane TC became higher than the in-plane TC. For example, at 30 wt% GNs, the through-plane TC was 25.57 W/(m·K), whereas the in-plane TC was only 2.39 W/(m·K), indicating strong anisotropy.

To analyze the anisotropic property of the TC for the composite sheets, we determined the ratio of the through-plane to in-plane TC (i.e., λ_⊥_/λ_∥_). As shown in Figure 5b, the λ_⊥_/λ_∥_ ratio for 1 wt% GNs–PFA composite was about 1.5, suggesting that the alignment of GNs in this nanocomposite was very weak. At 5 wt% GNs, the λ_⊥_/λ_∥_ ratio was approximately 5.9, indicating that a small number of GNs were vertically aligned in the nanocomposite sheet. As the GNs content increased to 10 wt%, the λ_⊥_/λ_∥_ ratio was about 10.1. For the GNs contents between 15 and 25%, the λ_⊥_/λ_∥_ ratio ranged from 14.2 to 15.5. According to Figure 4d,e, this high λ_⊥_/λ_∥_ ratio may be due to the high alignment and contact of GNs in the PFA matrix. At a GNs content of 30 wt%, the λ_⊥_/λ_∥_ ratio decreased to 10.7, which indicates that although the through-plane TC increased, the in-plane TC also increased owing to the increased compactness of GNs in the polymer. Overall, the results in Figure 5b demonstrate that when the GNs content in the composites was higher than 10 wt%, the TC exhibited strong anisotropy.

The through-plane TC of the PFA composite with 30 wt% GNs (25.57 W/(m·K)) was 9700% higher than that of pure PFA. Therefore, we compared the through-plane TC obtained in this work with the values reported in the literature for other GNs-based composites, which were fabricated by different methods, such as vacuum filtration [22], the liquid-crystal method [26], application of a magnetic field [27], and unidirectional freeze casting [28], among others [41,42,43,44,45,46,47]. The TCs of our GNs–PFA nanocomposites were higher than those reported in the literature. This may be because the degree of GNs alignment in the polymer matrix induced by hot pressing is much higher than that induced by the other methods as a result of the high pressure exerted on the nanocomposite surface during the hot-pressing process.

### 3.5. Heat Dissipation Capability of the GNs–PFA Nanocomposites

**Figure 6:**
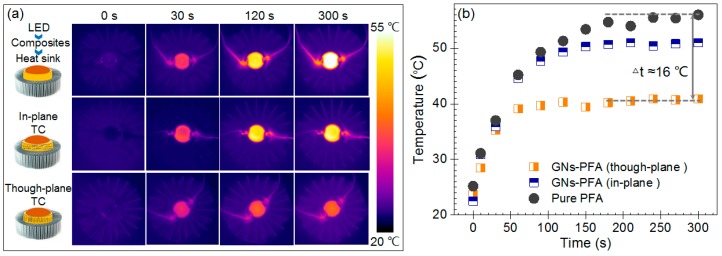
Infrared thermal images (**a**) and surface temperatures (**b**) as a function of time for high-power LEDs attached to pure PFA and the PFA nanocomposites with 30 wt% GNs (in-plane TC: Layer aligned GNs; through-plane TC: Vertically aligned GNs). In the experiment, the back surface of a high-power LED lamp was attached to a thin composite sheet, then a heat sink was connected to the bottom side of the composite as shown by the insets in (**a**).

To evaluate the application potential of the nanocomposites, we compared the heat-dissipation capability of the PFA composite sheet with 30 wt% GNs with that of pure PFA, by using them in the thermal management of a high-power LED. Note that, to make a direct comparison of in-plane and through-plane heat dissipation capability, as shown by the insets in Figure 6a, the composites containing layer aligned and vertically aligned GNs were both attached to the LEDs. Figure 6a shows the infrared thermal images for LEDs attached to pure PFA and the PFA composites after switching the LEDs on for 30, 120, and 300 s. The surface temperature of the LED was much lower when attached to the composite with vertically aligned GNs (through-plane TC) than the pure PFA. This clearly demonstrates the higher heat-dissipation capability of this composite than pure PFA.

Figure 6b shows the surface temperatures of the LEDs as a function of time. The surface temperature of the LED attached to pure PFA increased to approximately 56 °C within 150 s after it was switched on. While, the temperature of the LED attached to the composite with layer aligned GNs (in-plane TC) increased to approximately 51 °C within 100 s. By contrast, the temperature of the LED lamp attached to the composite with vertically aligned GNs (through-plane TC) increased to approximately 40 °C within 50 s and remained stable at this temperature. Thus, the temperature of the LED attached to the composite with vertically aligned GNs increased more quickly than that attached to pure PFA, and the maximum temperature was substantially lowered by 16 °C. This result demonstrates that the vertically aligned GNs increase heat dissipation capability of the composite in the direction of heat transfer.

### 3.6. Coefficient of Thermal Expansion (CTE) of the GNs–PFA Nanocomposites

**Figure 7:**
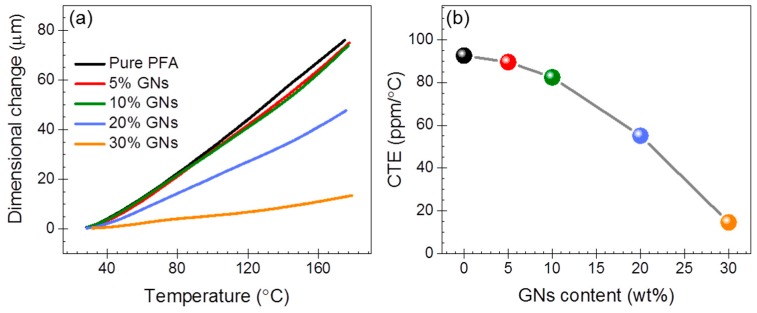
(**a**) Dimensional change of the samples as a function of temperature. (**b**) The determined CTE of nanocomposites.

As is known, poor dimension stability would directly influence the functionality of electronic devices [3,4,24]. To examine the dimension reliability, we determined CTE for the GNs–PFA nanocomposites. As shown in Figure 7a, the dimensional changes of the samples increased with the increasing temperature. Figure 7b depicts the determined CTE for pure PFA and the nanocomposites, which indicates that CTE decreased with the increasing GNs contents. In particular, pure PFA had a CTE of 92. 6 ppm/°C, while the values were 89.5, 82.4, 55.1 and 14.6 ppm/°C for PFA nanocomposites at the GNs contents of 5, 10, 20 and 30 wt%, respectively. The composite at 30 wt% GNs content possessed a low CTE. This is attributed to the well-ordered, and stacked aligned GNs stabilizing the whole framework of the composites.

In summary, the characterization of the thermal stability, microstructure, TC, heat dissipation, and CTE indicates that the 30 wt% GNs–PFA composite possessed excellent thermal stability, well vertically aligned GNs, high through-plane TC, and low CTE. Accordingly, considering that hot pressing and mechanical machining are both facile and low cost, we believe that our approach is effective for the large-scale production of GNs–PFA composites with high through-plane TCs.

## 4. Conclusions

In this work, we fabricated PFA composites with vertically aligned GNs via hot pressing followed by mechanical machining. We found that the GNs–PFA nanocomposites possessed good thermal stability. When the GNs content was higher than 10 wt%, the GNs were well aligned in the PFA composites owing to the hot pressing, and the through-plane TCs were about 10–15 times higher than the in-plane TCs. In addition, the through-plane TC of the PFA composite with 30 wt% GNs was 25.57 W/(m·K), which was 9700% higher than that of pure PFA. This study suggests that combining hot pressing with mechanical machining is a facile, low-cost strategy for large-scale production of GN–PFA nanocomposites with high through-plane TCs that can be used in various thermal-management applications.

## Supplementary Materials

The following are available online at https://www.mdpi.com/2079-4991/9/9/1320/s1, Figure S1. The optical densities of GNs dispersions obtained by exfoliating graphite in a series ethanol−water mixtures. Figure S2. The hot-pressing system used in this work. Table S1. The heat resistance index (THRI) for pure PFA and GNs–PFA nanocomposites.
