# Supplementary material for: Graphene−Perfluoroalkoxy Nanocomposite with High Through-Plane Thermal Conductivity Fabricated by Hot-Pressing
Source: Nanomaterials (Basel). 2019 Sep 15;9(9):1320. doi: 10.3390/nano9091320 (PMC6781095; doi:10.3390/nano9091320)
Supplement: Supplementary file 1 [file nanomaterials-09-01320-s001.pdf]

Supplementary Materials

Graphene−Perfluoroalkoxy Nanocomposite with High Through-Plane Thermal Conductivity Fabricated by Hot-Pressing

Xinru Zhang,[1,2] Xiaoyu Xie [1] Xinzhi Cai,[1] Zeyi Jiang [1,3,*] Ting Gao [1] Yujie Ren [4] Jian Hu [5] and Xinxin Zhang [1,3]

[1] School of Energy and Environmental Engineering, University of Science and Technology Beijing, Beijing 100083, China
[2] Beijing Engineering Research Center of Energy Saving and Environmental Protection, University of Science and Technology Beijing, Beijing 100083, China
[3] Beijing Key Laboratory for Energy Saving and Emission Reduction of Metallurgical Industry, University of Science and Technology Beijing, Beijing 100083, China
[4] China Energy Conservation and Environmental Protection Group, Beijing 100082, China
[5] China Energy Conservation and Environmental Protection Group National Machinery United Electric Power (Ningxia) Co., Ltd., Yinchuan 750011, China
* Correspondence: zyjiang@ustb.edu.cn; Tel.: 86-10-62334971

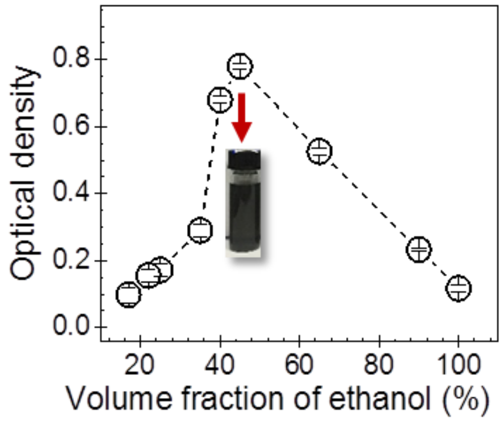

Figure S1. The optical densities of GNs dispersions obtained by exfoliating graphite in a series ethanol–water mixtures. It was found that the highest concentration of GNs dispersions was obtained in 45 vol% ethanol–water mixture. Therefore, in the study, the GNs were produced by exfoliating graphite in 45 vol% ethanol–water mixture.

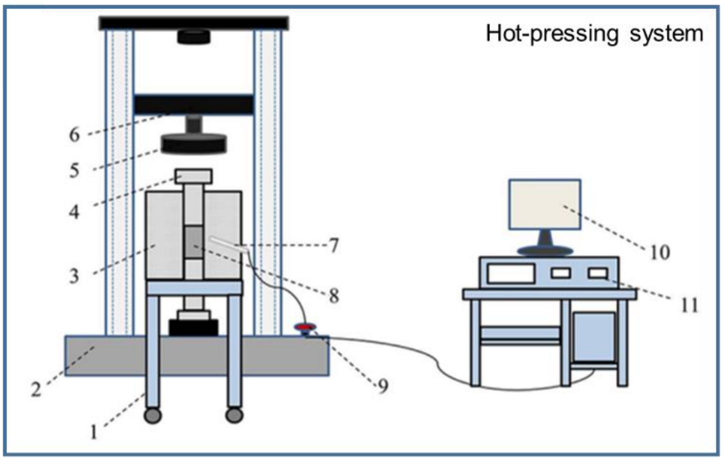

1 Base, 2 Electronic testing machine, 3 Heating furnace, 4 Pressure bar, 5 Indenter 6 Sensor, 7 Thermocouple, 8 Composite sample, 9 Switch, 10 Pressure control system 11 Temperature control system

Figure S2. The hot-pressing system used in this work.

Table S1. The heat resistance index (THRI) for pure PFA and the GNs–PFA nanocomposites.

Samples	Weight loss temperature (℃)		The heat resistance index（THRI）(℃)
	T_5	T_{30}	
Pure PFA	540.9	575.6	275.2
1% GNs	539.2	573.5	274.3
5% GNs	539.9	565.7	271.0
10% GNs	533.8	567.0	271.3
15% GNs	537.6	567.9	272.3
20% GNs	532.6	567.5	271.2
25% GNs	543.2	571.9	274.6
30% GNs	529.1	568.2	270.7

Note: T_5 and T_{30} are the temperature at 5% and 30% weight loss, respectively.
